# Supplementary material for: Analysis of the importance and priority of male and female players in mixed doubles table tennis
Source: PLoS One. 2026 Feb 10;21(2):e0339224. doi: 10.1371/journal.pone.0339224 (PMC12890131; doi:10.1371/journal.pone.0339224)
Supplement: Attachment — Submitted filename: Response to Reviewers.docx [file pone.0339224.s004.docx]

Reviewer #1: 
We sincerely thank Reviewer #1 for their valuable time and constructive feedback. These comments have greatly helped us improve the quality of our manuscript. We have carefully revised the manuscript in response to each of your comments. Our specific responses are as follows:

Terminology Consistency:
-The term "ping pong" in the abstract should be replaced with "table tennis" to maintain academic consistency and avoid confusion with sandpaper table tennis.

Reply: We thank the reviewer for the suggestion. The term "ping pong" has been replaced with "table tennis" .

-The interchangeable use of "players" and "athlete" throughout the paper should be standardized as well as "shot" and "stroke". "Players" and "stroke" are recommended for consistency.

Reply: We have revised the manuscript by replacing all instances of "athlete" with the standard term "player," as suggested. As for "shot" and "stroke", to our knowledge, while often used interchangeably, a 'stroke' mainly refers to the fundamental technique or form of the hit (e.g., forehand, backhand), whereas a 'shot' generally describes the tactical result and intention of the stroke in play (e.g., a winning shot, a defensive shot, the third shot). In accordance with the reviewer's comment, we have thoroughly checked the manuscript and made corrections to the relevant usage.

Conceptual Clarity of the Five-Phase Four-Mode Model:
-The justification for the "Five phase" is unclear. The literature cited (line 117) only supports three phases, not five. The authors must provide a stronger theoretical or empirical basis for defining five phases.

Reply: The description in the original text is as follows:“A mixed doubles match is divided into five phases by the Five-Phase and Four-Mode Model: P1, P2, P3, P4, and P5 based on previous phased evaluation theories [13, 22, 28, 29]”. The purpose of this formulation is to elucidate that this study developed a novel prioritization framework for mixed doubles table tennis by synthesizing experiences from various established segmentation methods. Specifically, our approach integrates insights from the Three-Phase Evaluation Method [13], the Eight-Round Three-Phase Method [22], the Four-Phase Method [28], and the Segmentation Method for Defensive Play (Chopping) [29]. In response to the reviewers' comments, we have revised the language accordingly, and the detailed theoretical interpretation of the model has been provided in the Discussion section.

-Defining each of the first four shots as separate phases is problematic. This approach conflates the concepts of "shot" and "phase," which have been distinctly defined in previous research. The authors should either revise this terminology or provide a compelling rationale for redefining these terms.

Reply: We sincerely thank the reviewer for raising this insightful and critical point regarding our terminology, specifically the conceptual distinction between a "shot" and a "phase" in our proposed model. We acknowledge that conventionally, a "phase" comprises multiple shots. Our deliberate departure from this convention is grounded in two fundamental rationales, central to the integrity and applicability of our model:

1. Adherence to Sport-Specific Characteristics: The primary principle of phased theory is to align with the unique characteristics of the sport to ensure both validity and practical utility. Our model is fundamentally derived from the distinct dynamics of mixed doubles table tennis, which are characterized by the critical importance of the first four shots, the strategic interplay between male and female athletes, and generally less refined coordination compared to singles play. The framework of designating P1 through P4 as distinct phases was constructed specifically to capture this essential structure of mixed doubles, and therefore, the core model architecture cannot be altered without compromising its foundational premise.

2. Conceptual Clarity and Model Cohesion: Within this framework, while P1 to P4 each technically represent a single "shot," they collectively represent the decisive, strategic stages that define the opening of a rally. In contrast, P5 aggregates all subsequent shots into a multi-shot phase. To present a clear, consistent, and usable model for readers and future practitioners, we have unified these strategic units under the term "phase." This culminates in the logically named "Five-Phase and Four-Mode Model." Furthermore, it is important to note that there are no universally mandated rules dictating the exact number of shots that must constitute a "phase." Our decision to isolate the first four shots as distinct phases intentionally serves to highlight their paramount importance in determining the outcome of a mixed doubles rally.

-The discussion section (lines 266-272) claims that the weak continuity in mixed doubles justifies treating each shot as a phase. However, this ignores the tactical coordination between teammates across shots (e.g., serve and third-shot attack). The authors should revise this section to align with established tactical analysis frameworks.

Reply: We appreciate the reviewer's comment. Our decision to segment the first four shots individually is precisely what grants our model its core analytical strength and flexibility.

Crucially, this design does not preclude consolidated analysis. The raw data from these phases can be directly merged to study combined phases like "serve-and-attack" or "receive-and-4th-shot-attack," aligning with traditional analytical frameworks. The distinct advantage, however, lies in the ability to perform granular, player-specific analysis for all four athletes—a level of detail that is lost in coarser models. This dual-capability ensures our framework is both comprehensive and adaptable.

We have revised the text (Line 265—268) to better articulate this flexibility.

Methodological Details:
-The consistency metrics mentioned (lines 108-111) need clarification. Specifically, which indicators (e.g., number of shots, technical actions) achieved a consistency value of 1? This must be explicitly stated.

Reply: We have revised the relevant expressions.

Data Presentation and Analysis:
-Table 2 requires layout optimization to enhance readability.

Reply: We have revised Table 2.

Statistical errors must be corrected:
-Line 221: The TDC1 values for male and female players should be stated as "6.5% and 2.5%, respectively."

Reply: We thank the reviewer for pointing this out. The error has been corrected in the manuscript.

-Lines 225-226: The "TDC order" values contain significant errors and require revision.

Reply: We thank the reviewer for pointing this out. The error has been corrected in the manuscript.

Citation formatting:
-Line 261: "(14)" should be replaced with "[14]."
-Line 266: "(6, 24)" should be replaced with "[6, 24]."

Reply: We thank the reviewer for pointing this out. The error has been corrected in the manuscript.

-Introduction: Remove "singles" from "table tennis singles competition" (line 54) to avoid redundancy with later uses of "singles."

Reply: We thank the reviewer for pointing this out. The error has been corrected in the manuscript.

Reviewer #2:

We sincerely thank Reviewer #2 for affirming our work and providing insightful and constructive feedback. Your high praise has greatly encouraged us, and your detailed revision suggestions have significantly enhanced the clarity and scientific rigor of our manuscript. We have revised the manuscript in response to each of your comments. Our specific responses are as follows:

-The authors of the paper relied on acceptable theoretical concepts in the introductory part and based on the aim of the paper, as well as on two valid approaches for calculating the efficiency of shots in table tennis (Zhang Liu and Tamaki et al), which are referred to in the part of the subtitle of the paper Computation of SR, LR, and E. I suggest that the mentioned subtitle be redefined, because the authors state abbreviations that do not appear in the text. The authors clearly define two goals that rely on a clear theoretical fashion.

Reply: We have revised the relevant subtitle in line 125.

Finally, we extend our sincere appreciation to the editors and reviewers for their thorough and constructive feedback, which has been instrumental in refining both the research and its presentation. In response to each of the suggestions raised, the authors have carefully revisited the manuscript and made extensive revisions to strengthen the depth of analysis, logical coherence, and clarity of expression. While every effort has been made to address the points raised, we recognize that there may still be aspects that can be further improved. We warmly welcome any additional comments and kindly request a re-evaluation of the revised manuscript. We apologize for any additional workload this process may have incurred and once again express our deepest gratitude. We look forward to the continued guidance of the editors and reviewers in helping this study reach its full potential.

Sincerely,

October 29, 2025
