# Supplementary material for: Analysis of the importance and priority of male and female players in mixed doubles table tennis
Source: PLoS One. 2026 Feb 10;21(2):e0339224. doi: 10.1371/journal.pone.0339224 (PMC12890131; doi:10.1371/journal.pone.0339224)
Supplement: Attachment — Submitted filename: Analysis of the importance and priority of ....docx [file pone.0339224.s003.docx]

### Manuscript title:

**“Analysis of the importance and priority of male and female players in mixed doubles table tennis”**

### Authors: Qing Yang, Muzi Li

### General assessment

The revised manuscript presents a clear and well-structured investigation into the tactical and technical dynamics of male and female players in mixed doubles table tennis. The study uses a novel analytical framework — the *Five-Phase and Four-Mode Model* — and applies appropriate quantitative techniques (multiple regression and total decision coefficient analysis) to describe role differentiation and performance contribution between male and female athletes.

Compared with typical analyses of singles or team play, this paper adds an original perspective that helps to better understand sex-specific interaction patterns in a highly coordinated sport context. The revised version is substantially improved in clarity, logic, and academic presentation.

Overall, the manuscript now demonstrates sufficient scientific rigor and conceptual coherence to be considered for publication after minor editorial polishing.

### **Strengths**

1. **Novel and sport-specific model**

The *Five-Phase and Four-Mode Model* represents a thoughtful and sport-specific approach to describing mixed doubles performance structure. The segmentation captures the asymmetrical tactical flow typical of male–female interactions during rallies.

2. **Strong empirical base**

The dataset, including 35 matches of top-50 ranked players, ensures ecological validity and relevance for elite-level performance analysis.

3. **Methodological improvement**

The use of multiple regression and TDC analysis is justified and clearly described in the current version. The authors provide sound reasoning for their analytical framework and terminology.

4. **Enhanced clarity and readability**

Language, structure, and figure formatting have been considerably improved. The paper is now coherent and easy to follow.

5. **Practical implications**

The translation of findings into applied recommendations (e.g., the “lady-first” serve strategy) provides practical value for coaches and performance analysts.

### **Minor comments and suggestions**

1. **Terminology consistency**

The revised manuscript successfully standardizes “players” instead of “athletes.”

However, the distinction between *“shot”* and *“stroke”* could still be made explicit in the methods section (a brief sentence defining both terms would improve clarity for readers unfamiliar with racket sport terminology).

2. **Figures and tables**

Figures are now well-structured, but Figure 2 would benefit from slightly larger axis labels

3. **Statistical interpretation**

The explanation of “performance impact percentage” is now clearer, but it would be valuable to briefly remind readers in the Discussion of how this metric should be interpreted in practical terms (e.g., effect magnitude vs. tactical significance).

4. **English style and phrasing**

The English is of generally high quality, though a brief professional proofreading would further enhance fluency (especially sentence transitions and punctuation consistency).

### **Recommendation**

**Minor Revision**

The manuscript presents a well-designed and methodologically sound study with meaningful practical implications. The revisions have effectively addressed the earlier issues raised by reviewers.

Only minor editorial refinements are recommended before acceptance — mainly small clarifications and stylistic polishing.

The revised manuscript demonstrates consistent statistical reporting and clear terminology, reflecting careful integration of previous reviewer feedback.

**Recommendation: Accept after minor revision.**
