# Supplementary material for: Analysis of the importance and priority of male and female players in mixed doubles table tennis
Source: PLoS One. 2026 Feb 10;21(2):e0339224. doi: 10.1371/journal.pone.0339224 (PMC12890131; doi:10.1371/journal.pone.0339224)
Supplement: Attachment — Submitted filename: Response_to_Reviewers_auresp_2.docx [file pone.0339224.s005.docx]

Reviewer:

Thank you very much for your valuable comments and suggestions. We appreciate and are greatly motivated by the kind comments that recognize the potential of our work. We have done our best to address all the concerns raised and revised the paper accordingly. The detailed changes are listed as follows：

**Minor comments and suggestions**

**1. Terminology consistency**

The revised manuscript successfully standardizes “players” instead of “athletes.”

However, the distinction between “shot” and “stroke” could still be made explicit in the methods section (a brief sentence defining both terms would improve clarity for readers unfamiliar with racket sport terminology).

Reply: We thank the reviewer for the suggestion. A short sentence of explanation was inserted at line 108-110 of the Methods section for clarity.

**2. Figures and tables**

Figures are now well-structured, but Figure 2 would benefit from slightly larger axis labels.

Reply: We thank the reviewer for pointing this out. Figure 2 has been revised as requested, and the updated figure file has been submitted separately.

**3. Statistical interpretation**

The explanation of “performance impact percentage” is now clearer, but it would be valuable to briefly remind readers in the Discussion of how this metric should be interpreted in practical terms (e.g., effect magnitude vs. tactical significance).

Reply: We appreciate this suggestion. As recommended, we have added explanatory content in the Discussion section (lines 327-333) to clarify this point.

**4. English style and phrasing**

The English is of generally high quality, though a brief professional proofreading would further enhance fluency (especially sentence transitions and punctuation consistency).

Reply: The text has been carefully revised/edited throughout the manuscript for clarity and expression. Please refer to the revised manuscript for the changes.

Finally, thank you again for your time and assistance. We hope that this revised version addresses all the concerns of the reviewers. We look forward to the continued guidance of the editors and reviewers in helping this study reach its full potential.

Sincerely,

December 26, 2025
